# Acute myeloid leukemia with adult atopic dermatitis as first manifestation: A case report

**DOI:** 10.1097/MD.0000000000016362

**Published:** 2019-08-09

**Authors:** Wei-Wei Ma, Lorna Martin Kasyanju Carrero, Xu-Feng Yin, Hou-Fang Liu, Bing-Rong Zhou

**Affiliations:** Department of Dermatology, The First Affiliated Hospital of Nanjing Medical University, Nanjing, Jiangsu, China.

**Keywords:** atopic dermatitis, acute myeloid leukemia

## Abstract

**Rationale:**

Atopic dermatitis (AD) is a chronic recurrent dermatitis with profound itching, which could be the first manifestation of acute myeloid leukemia (AML).

**Patient concerns:**

A 53-year-old Chinese man suffered a 6-month history of systemic symmetrical dermatitis, accompanied with profound itching. The patient was diagnosed as “eczema” in several hospitals, and the effects of antihistamine and topical steroid creams were poor. Nocturnal sleep was seriously affected by aggravating pruritus. Laboratorial examination was compatible with AML-M4.

**Diagnoses:**

AML-M4 with AD as first manifestation.

**Interventions:**

IA regimen (ayninen and cytarabine) were used in induction chemotherapy. However, the patient did not achieve complete remission, and although his rash had improved, he still experienced severely general body itching. On the seventh day of chemotherapy, the patient entered the period of granulocyte deficiency with infection.

**Outcomes:**

The patient died due to septic shock after chemotherapy.

**Lessons:**

The case strengthens the awareness of AML with AD as first manifestation and raises oncological vigilance in patients with AD refractory.

## 1 Introduction

Studies reported that acute myeloid leukemia (AML) had a wide range of cutaneous manifestations in clinical and morphological findings such as nodular lesions and plaques. These manifestations could be divided into specific and nonspecific.^[1,2]^ One meta-analysis study indicated that there was an inverse association between atopy/allergies and childhood acute lymphocytic leukemia (ALL), while no association was observed for AML or leukemia overall.^[3]^ And there were no reports of AML with atopic dermatitis (AD) as first manifestation. Herein we report a 53-year-old man who was finally diagnosed as AML, with AD as a first manifestation.

## 2 Case report

**Figure 1:**
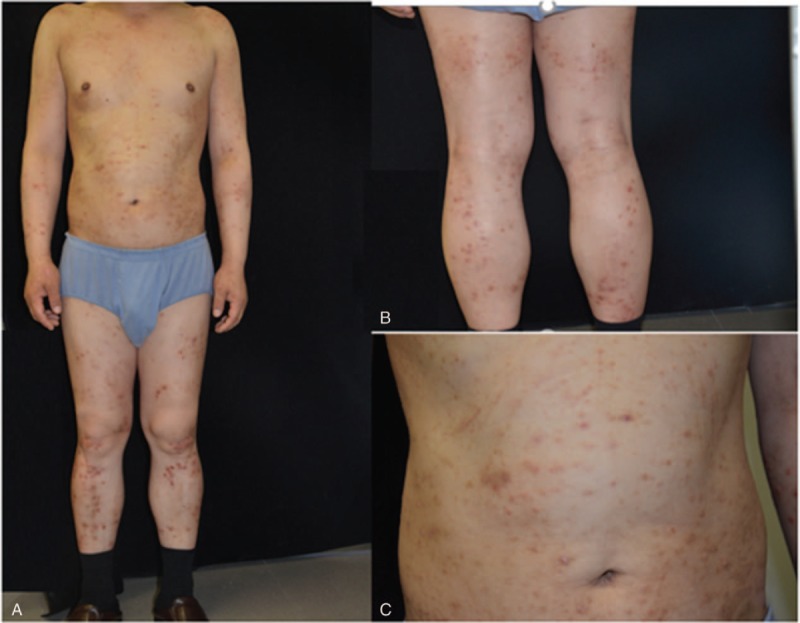
(A) Multiple discrete, erythematous papules and scales on the trunk. (B and C) Closer views of the erythematous papules and scales on the lower extremities and abdomen.

A 53-year-old Chinese male visited the hospital due to suffering systemic symmetrical dermatitis for more than 6 months (Fig. 1). The patient was diagnosed as “eczema” in several hospitals, and the effects of antihistamine and topical steroid creams were poor. Nocturnal sleep was seriously affected by progressive aggravating pruritus. No abnormal index was found in previous laboratory studies. The patient reported allergic rhinitis diagnosed previously, and he had a long history of exposure to chemical substances as an engineer, while his children had a history of allergic dermatitis. Laboratory study showed a total serum immunoglobulin E (IgE) level of 604.61 KU/L, a leukocyte count of 3.7 × 10^9^/L with 35.50% lymphocytes, 41.40% neutrophils, 8.3% monocytes, 14.50% eosinophil, hemoglobin of 144 g/L, and platelet count of 155 × 10^9^/L. According to the Chinese Criteria for AD proposed by Zhang et al,^[4]^ the patient was diagnosed as AD and was treated with antihistamines, *Tripterygium wilfordii*, and the dermatitis was treated with oral prednisone (20 mg/d). After 1 and a half months, he felt consciously relieved of itching and the rash was relieved. But there was still development of new rashes.

**Figure 2:**
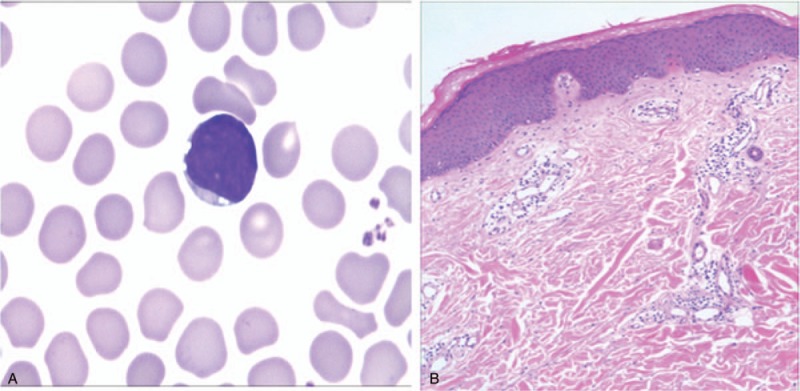
(A) The peripheral blood smear showed Auer corpuscles in certain cells. (B) Incisional biopsy of erythematous papules showed hyperkeratosis accompanied by parakeratosis, edema of acanthosis thickened, and dilated capillary with infiltration of mononuclear both in superficial and medial layer, H&E stain 100×. H&E = hematoxylin and eosin.

Therefore, the patient visited our hospital again. Laboratory study showed a leukocyte count of 8.0 × 10^9^/L with 46.70% lymphocytes, 21.90% neutrophils, 15.40% monocytes, hemoglobin of 134 g/L, and platelet count of 190 × 10^9^/L. A peripheral blood smear showed the normal total leukocyte count with 33% blasts, and Auer corpuscles can be seen in certain cells (Fig. 2A). The bone marrow smear indicated the active granulocyte proliferation, the original granulocyte type I + II accounting for 50.4% with the Auer corpuscles in cells and the promyelocyte accounting for 0.8%. No chromosomal abnormalities or fusion genes were detected. Immunohistochemistry of the skin biopsy showed hyperplasia of lymphoid tissue, CD34(−), CD117(−), MPO (+), CD43(++), CD20(±), CD3(+), and Ki-67(+ scattered). And combined with the hematoxylin and eosin stain (HE) section (Fig. 2B), the case was considered chronic inflammation of the dermis with lymphadenosis. The diagnosis of AML subtype M4 was made.

According to National Comprehensive Cancer Network Guidelines,^[5]^ standard dose of IA regimen (ayninen and cytarabine) was given. The patient had a fever after receiving chemotherapy for 3 days; we reduced the dose of ayninen to continue chemotherapy. After anti-allergic and chemotherapy, the rash was flatter than before, but the skin was too itchy to bear. On the seventh day of chemotherapy, the patient entered a period of granulocyte deficiency and went into septic shock. Finally, the patient was transferred to the ICU and died in June 2018.

## 3 Discussion

Leukemia has a wide range of cutaneous manifestations in clinical and morphological findings, which are traditionally divided into nonspecific lesions and specific lesions.^[6]^ The specific lesions called leukemia cutis (LC) are considered as malignant lesions, which are localized or disseminated infiltrations of the skin by leukemic cells. And it may involve all layers of the skin.^[7]^ LC may often present with nodular lesions and plaques. While the clinical manifestations like erythematous macules, blisters, and ulcers are rare, and they may appear alone or in combination.^[1]^ LC has been described in patients with AML, chronic myelogenous leukemia, myelodysplastic syndromes, and myelodysplastic and lymphoproliferative diseases. At present, many reports about AML–LC have been published.^[8–11]^ And LC was somewhat higher in AML-M4 and AML-M5 than in other types of leukemia.^[2]^

Nonspecific lesions showed varied morphology and could be difficult to distinguish from specific cutaneous lesions, both clinically and histopathologically.^[2]^ There were about 30% of cases of AML with nonspecific lesions.^[7]^ To the best of our knowledge, nonspecific lesions associated with AML have been reported already.^[2,12]^ Rao et al^[13]^ proposed that nonspecific lesions were with a wide spectrum of etiologies, including infection, drug rash, purpura, graft versus host disease, vasculitis, the Sweet syndrome, and intraepidermal blistering disorder. Several pro-inflammatory cytokines such as tumor necrosis factor-α and interleukin-1 elevated significantly in leukemia,^[14,15]^ we presumed that these elevated cytokines may be associated with nonspecific lesions. The origins of these cytokines are unknown, but these proteins may be secreted by leukemic cells in the bone marrow.^[16]^

AD is a chronic recurrent dermatitis with profound itching. Zhang et al^[4]^ proposed 3 features as the criteria for AD: eczema for more than 6 months; personal and/or family history of atopic diseases; elevated total serum IgE level and/or positive allergen-specific IgE and/or eosinophilia.^[4]^ One meta-analysis indicated that there was an inverse association between atopy/allergies and childhood ALL while no association was observed for AML or leukemia overall.^[3]^ Compared with other AML patients, the case we reported was finally diagnosed as AML, with AD as a first manifestation and there was no report about this before.

Whether there is an inevitable relationship between AML and AD remains unclear. One hypothesis is that AD may be a hypersensitive reaction of the skin immune system to leukemia cells and presents as a significant increase in total serum IgE level and/or eosinophils. One article revealed that the increased numbers of eosinophils at all stages of maturation in AML-M4 could be associated with abnormal eosinophil precursors.^[17]^ Further research is needed to investigate the pathogenic relevance between AML and the elevated level of eosinophils in AD.

The case of AML-M4 with AD as a first manifestation would shed light on both clinical work and research in the future. AD may be the first presentation of AML. Clinically, leukemia with rash as first clinical manifestation is easily misdiagnosed and missed in clinical. Therefore, with a patient of AD, we should take leukemia as consideration and perform a thorough work-up. Furthermore, AD may suggest a poor prognosis for patients with AML. So, prompt and accurate identification of AD accompanied by AML is crucial for timely management.

As an engineer, the patient had a long history of exposure to chemical substances and maybe this is the direct cause of the dermatitis. Perhaps this plays a role in the pathogenesis of leukemia. But it needs further confirmation.

In brief, we showed a rare case of AML-M4 associated with AD, which strengthens the awareness of AD associated with AML and raises vigilance against oncology in patients with AD.

## Author contributions

**Data curation:** Xu-Feng Yin, Hou-Fang Liu.

**Writing – original draft:** Wei-Wei Ma.

**Writing – review & editing:** Wei-Wei Ma, Lorna Martin Kasyanju Carrero, Bing-Rong Zhou.
